# Clinical factors associated with valgus knee deformities in patients with multiple osteochondromas

**DOI:** 10.1097/MD.0000000000042359

**Published:** 2025-05-09

**Authors:** Kan Ito, Yoshihiro Nishida, Kunihiro Ikuta, Hiroshi Urakawa, Tomohisa Sakai, Hiroshi Koike, Kazuki Nishida, Shiro Imagama

**Affiliations:** a Department of Orthopedic Surgery, Nagoya University Graduate School of Medicine, Nagoya, Aichi, Japan; b Department of Rehabilitation, Nagoya University Hospital, Nagoya, Aichi, Japan; c Department of Advanced Medicine, Nagoya University Hospital, Nagoya, Aichi, Japan; d Rare Cancer Center, Nagoya University Hospital, Nagoya, Aichi, Japan.

**Keywords:** femorotibial angle, genu valgum, multiple osteochondromas, neck-shaft angle, osteochondromatosis, valgus knee deformities

## Abstract

Multiple osteochondromas (MO) occur in approximately 1 in 50,000 people/yr. One in 3 patients with MO will develop valgus knee deformity (VKD), but the predictive factors for VKD are unclear. The purpose of this study was to examine the factors associated with VKD in patients with MO. From January 2003 to December 2018, 64 patients with MO visited the Nagoya University Hospital for the 1st time. Thirty-three patients with 66 limbs were sequentially included in the study after excluding 12 patients with a history of lower extremity surgery, 15 patients whose knee X-rays were unavailable, and 4 patients whose age at the last examination was <7 years. Limbs with femorotibial angle (FTA) ≥ 175° were defined as the normal group (Group N) and limbs with FTA < 175° as the valgus group (Group V), and clinical factors collected retrospectively from the medical records were compared between the 2 groups. The initial and final X-rays were compared in a subgroup analysis of 8 patients whose initial examination was <10 years old and who were followed for more than 5 years. Twenty-four males and 9 females with a median age of 17 years at the last X-rays were included in the study. The mean follow-up period was 43 ± 53 months, and the median FTA was 174.5°. Group N consisted of 32 limbs and Group V consisted of 34 limbs. Multivariate analysis was performed using the 5 factors with *P*-values <.15 in the univariate analysis of comparison between the 2 groups, and only medial proximal tibial angle showed significant differences (*P* < .001). In the subgroup analysis, multivariate analysis showed that the femoral neck-shaft angle showed significant differences between the 2 groups at the initial evaluation (*P* < .001). Our study suggests that medial proximal tibial angle is associated with VKD in patients with MO. Small neck-shaft angle was significantly associated with VKD, even before it became obvious. In order to study how VKD is formed, imaging of the hip and ankle joints and X-rays of the entire lower extremity should be performed in more cases.

## 1. Introduction

Hereditary or solitary multiple osteochondromas (MO) occur in approximately 1 in 50,000 people/yr, with a higher prevalence in males than females.^[1]^ MO is characterized by the presence of multiple benign cartilage-capped bone tumors (osteochondromas) that develop near the growth plates of long bones. The condition is caused by germline mutations in the *EXT1* and *EXT2* genes, which encode glycosyltransferases involved in heparan sulfate biosynthesis.^[2,3]^ Among the various skeletal complications associated with MO, valgus knee deformity (VKD) is one of the most common and functionally important complications. VKD occurs in 1 in 3 patients with MO, and the size and number of osteochondromas around the knee are associated with the development of VKD.^[4–6]^ Clement et al reported that the presence of a tumor in the distal femur is a predictor of VKD.^[7]^ While these studies provide valuable insights, they predominantly focus on localized knee-related factors based on radiographic imaging. Madoki et al focused on the presence of distal as well as proximal intertibio-fibular exostoses and reported that their presence is a risk factor for VKD,^[8]^ but no reports have yet focused on the entire lower extremity. Evaluating the relevant factors can help identify patients at high risk for VKD and avoid the development of deformity by performing minimally invasive epiphysiodesis during skeletal growth. The aim of this study was to examine factors associated with VKD in patients with MO, which may lead to early identification and timely treatment of VKD.

## 2. Materials and methods

From January 1, 2003 to December 31, 2018, 64 patients with MO visited Nagoya University Hospital for the 1st time. Twelve patients with a history of lower extremity surgery, 15 patients with no knee X-rays available, and 4 patients with ages <7 years at the last examination were excluded from the study. Thus, 33 patients with 66 limbs were included in the analysis. Twenty-six patients were inherited, 6 were not-inherited, and 1 was unknown.

**Figure 1.**
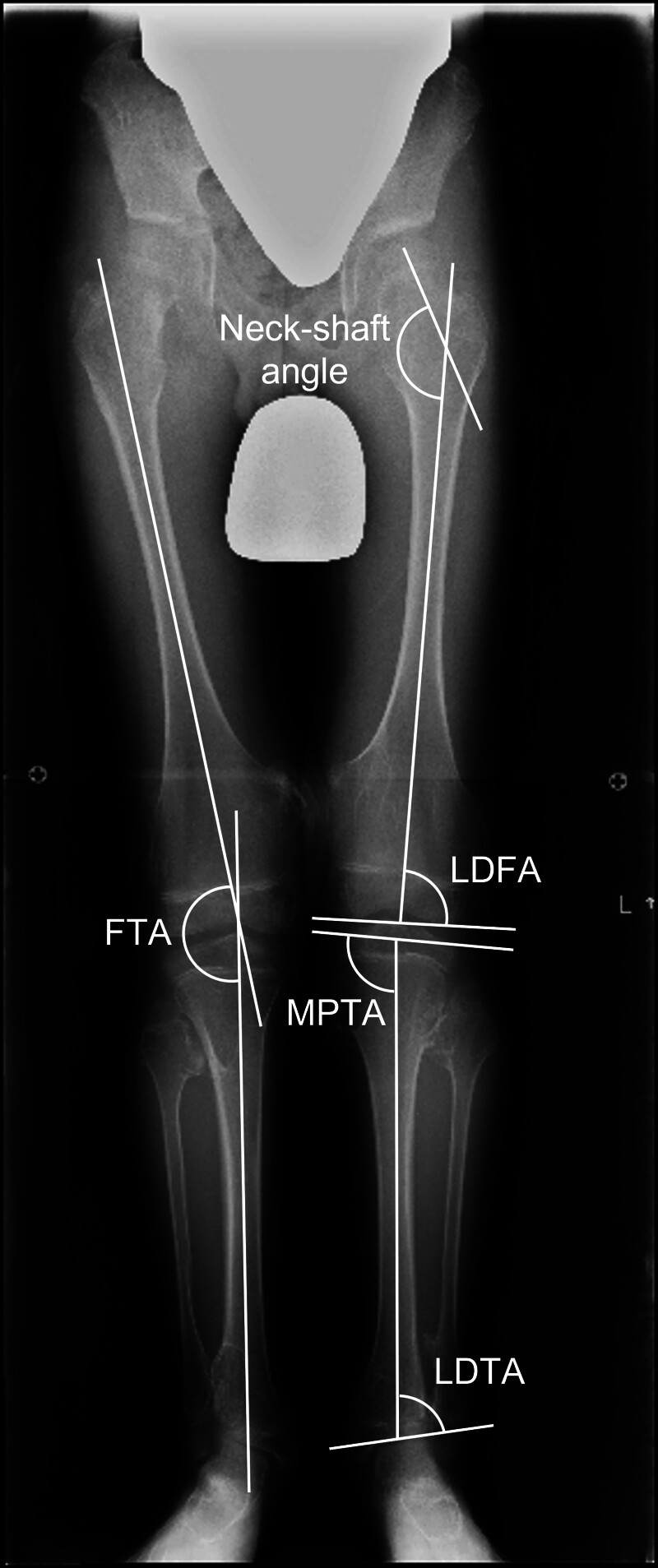
Graphical illustration of bone morphological angles in an X-ray of the entire length of the lower extremity. Neck-shaft angle was calculated from the femoral long axis and femoral neck axis in the frontal view of the X-ray image. FTA is the angle formed between the long axis of the femur and the long axis of the tibia. LDFA is the angle between the long axis of the femur and the joint line of the distal femur. MPTA is the angle between the long axis of the tibia and the joint line of the proximal tibia. LDTA is the angle between the long axis of the tibia and the joint line of the distal tibia. FTA = femorotibial angle, LDFA = lateral distal femoral angle, LDTA = lateral distal tibial angle, MPTA = medial proximal tibial angle.

The diagnosis of MO was based on radiological findings, while CT, MRI, and pathology results were used as supplemental tools. The following clinical characteristics were retrospectively collected from the medical records: gender, age at last X-rays, heredity, femorotibial angle (FTA), lateral distal femoral angle (LDFA), medial proximal tibial angle (MPTA), lateral distal tibial angle (LDTA), femoral neck-shaft angle, center-edge (CE) angle of Wiberg, Sharp angle, tumor localization (proximal femur, medial or lateral distal femur, medial or lateral proximal tibia, proximal fibula, proximal or distal tibiofibular joint), tibia/fibula length ratio, and femur/tibia length ratio. LDFA, MPTA, and LDTA were not measured using the mechanical axis of the lower extremity, but rather anatomical angles calculated from the long axis of the femur and tibia (Fig. 1). Because it is difficult to determine whether the tumor is medial or lateral in the proximal femur, we considered only the presence or absence of the tumor. Each radiological finding was measured on anteroposterior X-ray images of the hip, knee, and ankle joints. X-rays of the entire lower extremity length were taken in the standing position. All measurements were performed by a single experienced investigator. To enhance reliability, the investigator underwent a training phase, conducting repeated measurements until intra-observer consistency was achieved. For patients who underwent surgery during the follow-up period, the examination immediately before surgery was considered the last examination.

VKD is defined using FTA, but there isn’t a completely uniform definition across all sources. Following a previous report by Porter et al,^[9]^ we defined a knee with FTA of <175° as VKD. Limbs were classified into 2 groups, as follows: normal group (Group N), FTA ≥ 175° and valgus group (Group V), FTA < 175°.

Patient characteristics were summarized using mean and standard deviation for continuous variables and frequency and percentage for categorical variables. To compare each clinical factor in Groups N and V, a chi-square test was performed for categorical variables and a Mann–Whitney *U* test for continuous variables. A multiple regression analysis was performed using factors with *P*-values <.15 in the univariate analysis, adjusting for potential confounders.

In a subgroup analysis, to evaluate the development of VKD over time during growth, 8 patients (eight limbs each in the N and V groups) who were diagnosed at <10 years old and followed for more than 5 years were compared. Age, heredity, FTA, LDFA, MPTA, neck-shaft angle, and CE angle, Sharp angle were compared from the initial and final X-rays. Due to the lack of radiographic images of the hip and lower leg, neck-shaft angle, CE angle, and Sharp angle at the last examination were analyzed in 7 limbs per group and the tibia/fibula length ratio was analyzed in 5 limbs from Group N and 7 limbs from Group V. For the same reason, tibia/fibula length ratios at the time of the initial examination were analyzed in 3 limbs in Group N and 5 limbs in Group V. Statistical analysis was performed in the same method as for the overall analysis described above.

All statistical analyses were performed using EZR (Saitama Medical Center, Jichi Medical University, Saitama, Japan).^[10]^ Given the exploratory nature of this study, which aimed to assess potential associations, *P*-values were not adjusted for multiple comparisons to avoid missing potential signals. Statistical significance was set at *P* < .05.

### 2.1. Ethical considerations

Our institutional review board approved this study (approval number: 2015-0358). In this approval, the need for informed consent was waived by our institution because of the retrospective design of the study based on anonymous data. This research was conducted in accordance with the principles set out in the Declaration of Helsinki.

## 3. Results

Patient characteristics are shown in Table 1. The study population consisted of 24 males and 9 females with a mean and median age at the last X-rays of 23 ± 14 and 17 (10–66) years. The mean follow-up period was 44 ± 52 months. Comparisons of the clinical factors between Groups N and V are also shown in Table 1. Group N included 32 limbs, and Group V included 34 limbs. The results of the univariate analyses showed significant differences in LDFA, MPTA, CE angle, and Sharp angle between the 2 groups (*P* = .01, *P* < .001, *P* = .007, and *P* = .002, respectively). Neck-shaft angle tend to be related to VKD (*P* = .09). In the multivariate analysis performed using these 5 factors, only MPTA showed significant differences (*P* < .001).

**Table 1:** Patient characteristics and comparison of clinical factors between Groups N and V.

Variables		All	Group N 32 limbs	Group V 34 limbs	Univariate			Multivariate
					*P*-value	Regression coefficient	95%CI	*P*-value
Sex (male)		24 (73%)	66%	79%	.27			
Age* (yr)		23 ± 14	23 ± 15	22 ± 13	.69			
Heredity (familial)	(32 patients)	26 (81%)	87%	76%	.35			
Follow-up period* (mo)		44 ± 52	40 ± 54	48 ± 51	.54			
FTA* (°)		172.7 ± 5.9	177.5 ± 2.2	168.2 ± 4.6	–			
LDFA* (°)		81.5 ± 3.9	82.7 ± 3.4	80.3 ± 4.0	.01	−0.031	(−0.062, 0.000)	.051
MPTA* (°)		90.6 ± 5.2	87.9 ± 4.2	92.6 ± 4.7	<.001	0.055	(0.034, 0.077)	＜.001
LDTA* (°)	(41 limbs)	82.8 ± 5.3	81.7 ± 4.0	84.1 ± 6.3	.21			
Neck-shaft angle* (°)	(50 limbs)	143.6 ± 7.5	145.6 ± 8.3	141.9 ± 6.3	.09	−0.019	(−0.028, 0.004)	.148
CE angle* (°)	(52 limbs)	32.3 ± 8.1	29.2 ± 7.3	35.2 ± 7.8	.007	0.002	(−0.020, 0.023)	.883
Sharp angle* (°)	(52 limbs)	42.3 ± 4.6	44.3 ± 4.2	40.5 ± 4.2	.002	−0.032	(−0.072, 0.008)	.115
Locations								
Proximal femur	(52 limbs)	47 limbs (90%)	88%	93%	.66			
Distal medial femur		60 limbs (91%)	91%	91%	>.99			
Distal lateral femur		55 limbs (83%)	78%	88%	.33			
Proximal medial tibia		62 limbs (94%)	94%	94%	>.99			
Proximal lateral tibia		54 limbs (82%)	78%	85%	.53			
Proximal fibula		62 limbs (94%)	91%	97%	.35			
Proximal tibiofibular joint		54 limbs (82%)	82%	82%	>.99			
Distal tibiofibular joint	(41 limbs)	36 limbs (88%)	82%	92%	.63			
Tibia/fibula length ratio*	(26 limbs)	1.06 ± 0.03	1.06 ± 0.03	1.07 ± 0.03	.3			
Femur/tibia length ratio*	(22 limbs)	1.20 ± 0.05	1.21 ± 0.05	1.20 ± 0.06	>.99			

CE = center-edge, CI = confidence interval, FTA = femorotibial angle, LDFA = lateral distal femoral angle, LDTA = lateral distal tibial angle, MPTA = medial proximal tibial angle.

* mean ± SD.

In order to examine early risk factors associated with the VKD development, a subgroup analysis of 16 limbs in 8 patients (8 limbs per group) was performed (Table 2). Multiple regression analysis of the comparison between the 2 groups at the initial evaluation showed that only the neck-shaft angle had a significant difference (*P* < .001). The mean neck-shaft angle on X-ray at the initial examination was 155.8° in Group N and 141.6° in Group V. While the neck-shaft angle was significantly smaller in Group V, the mean FTA at the initial examination was 179.7° in Group N and 173.9° in Group V, with no significant difference between the 2 groups.

**Table 2:** Subgroup analysis of predictors of valgus knee deformity in patients with multiple osteochondromas.

Variables	8 patients (16 limbs)	Group N 8 limbs	Group V 8 limbs	Univariate			Multivariate
				*P*-value	Regression coefficient	95%CI	*P*-value
At last examination							
FTA* (°)	171.6 ± 6.7	177 ± 1.1	166.3 ± 5.4	–			
LDFA* (°)	83.5 ± 3.6	84.4 ± 2.9	82.6 ± 3.9	.34			
MPTA* (°)	93.6 ± 4.1	92.4 ± 4.0	94.8 ± 3.8	.53			
Neck-shaft angle* (°)	146.6 ± 6.6	148.9 ± 8.5	144.3 ± 3.7	.52			
CE angle* (°)	25.4 ± 5.0	24.1 ± 3.0	26.6 ± 6.2	.7			
Sharp angle* (°)	47.3 ± 3.1	48.4 ± 1.9	46.1 ± 3.6	.22			
At initial examination							
FTA* (°)	176.8 ± 5.6	179.7 ± 3.7	173.9 ± 5.7	.12	0.015	(−0.006, 0.037)	.146
LDFA* (°)	87.4 ± 3.3	85.6 ± 2.7	89.1 ± 2.9	.06	0.023	(−0.010, 0.056)	.156
MPTA* (°)	87.5 ± 4.4	86.9 ± 1.6	88.1 ± 5.9	.63			
Neck-shaft angle* (°)	148.1 ± 8.0	155.8	141.6	.002	−0.059	(−0.075, −0.043)	＜.001
CE angle* (°)	17.6 ± 5.7	15.3 ± 5.9	20 ± 4.3	.13	0.006	(−0.014, 0.027)	.49
Sharp angle* (°)	43.9 ± 7.0	45.9 ± 8.4	42 ± 4.3	.67			

CE = center-edge, CI = confidence interval, FTA = femorotibial angle, LDFA = lateral distal femoral angle, LDTA = lateral distal tibial angle, MO = multiple osteochondromas, MPTA = medial proximal tibial angle, VKD = valgus knee deformity.

* mean±SD.

## 4. Discussion

One in 3 patients with MO develops VKD.^[4–6]^ Patients with VKD often complain of walking difficulty, limited range of motion of the knee joint, and poor visual appearance. Patients with severe VKD can be treated with temporary hemiepiphysiodesis during the skeletal growth period.^[11–14]^ Early detection and predicting the onset of VKD is important for timely intervention.

Knee deformity in patients with MO is associated with the size and number of osteochondromas around the knee during skeletal growth from 10 to 14 years.^[6]^ Nawata et al reported that increased MPTA is associated with VKD,^[6]^ and Liu et al reported that LDFA is also associated with VKD.^[15]^ Tumor localization in the distal femur^[7]^ or proximal and distal tibiofibular joints^[16]^ have also been reported to be associated with VKD. However, these parameters are based on imaging findings around the knee, and no multiple factor analyses including hip and ankle joints. This is the 1st analysis of factors associated with VKD in patients with MO that includes factors involving the total length of the lower extremity. The results of the present study revealed that MPTA is associated with VKD. On the other hand, the results of the present study did not indicate that tumor localization contributes to the development of VKD, as has been reported previously.^[7,8,16]^ And we would like to emphasize that no studies focused on bone morphological variables of VKD development in patients with MO before the deformity occurs. Although no definitive conclusions can be made due to the small number of cases, this study suggested neck-shaft angle is associated with the onset of VKD from an early age.

The following mechanisms for the development of VKD can be speculated from the results of the present study. If the neck-shaft angle is reduced early, it will result in coxa vara, and VKD may develop in order to compensate for the load axis of the entire lower limb.

Tumor size and location vary from case to case; thus, a variety of factors influence each other. In the present study, univariate analysis showed that 5 factors, LDFA, MPTA, and cervical axis angle, CE angle, and Sharp angle, had *P*-values <.1, suggesting that many factors are associated with VKD in a complex mechanism. Similarly, in the subgroup analysis, if the number of cases was larger, there might have been significant differences in several factors. To investigate the complex interactions of these multiple factors, imaging of the entire lower extremity, including the hip and ankle joints, is necessary. However, in practice, X-rays are often only taken around the knee area. Furthermore, detailed evaluations of the hip or ankle joints using CT or MRI were rarely performed. In the hip joint, the presence, size, and location of tumors in the proximal femur are difficult to determine, based only on X-rays. To elucidate the pathophysiology of lower limb deformity in patients with MO, a detailed imaging evaluation of the entire lower extremity is necessary. However, radiation exposure is a problem in CT, and both CT and MRI are costly; thus, these examinations should be performed at a minimal frequency.

Age limitations were set in the inclusion and exclusion of patients in the present study. Patients who were <7 years of age at the last examination were not included to exclude the possibility of physiologic valgus knee as much as possible. In addition, the subgroup analysis was limited to patients who were <10 years old at the 1st visit, because Nawata et al reported that abnormalities in LDFA and MPTA begin at approximately 10 years of age.^[6]^

There are several limitations to the present study. First, because it was a retrospective study, an insufficient number of cases were included in the analysis, imaging information was lacking, and the mean follow-up period was relatively short. Only a few cases could be followed for more than 5 years before and after the skeletal growth period; thus, the number of cases was not sufficient to determine truly meaningful associated factors. In addition, several cases had missing imaging information for the entire lower extremity, including the hip and ankle joints. For 36 of the 66 limbs, not all data were available due to missing measurements. No significant difference was found between the presence or absence of VKD and the presence or absence of missing measurements using a chi-square test (*P*-value = 1.0). Therefore, although missing measurements do not appear to have a significant effect, they may have a potential effect that should be examined in future studies. Moreover, if MRIs had been taken in most of the cases, the variables of the lesion, such as the size of the cartilage cap and its distance from the epiphyseal line, could have been examined. Second, patients with MO may have a poor range of motion, but the present study did not include range of motion or functional scores. Third, the VKD phenotype is more severe in patients with *EXT1* mutations than those with *EXT2* mutations,^[9,17,18]^ but the present study did not examine genetic mutation types. Fourth, there is the possibility that the radiographic conditions are not consistent. In particular, neck-shaft angle may be greatly affected by the degree of internal/external rotation of the lower limb at the time of radiography. For example, a difference of 20° of internal/external rotation could potentially result in a measurement error of 10 to 14° of neck-shaft angle. It is necessary to distinguish the appropriate imaging method for each measurement. The FTA measurement requires an X-ray of the entire length of the lower extremity in the standing position, and the hip, knee, and ankle joints require their own dedicated and accurate frontal images.

Based on the results of the present study, our recommendations for lower extremity management in patients with MO are presented. Because VKD develops around the age of 10 years, it is recommended that MO patients undergo regular X-ray examinations before the age of 10 years to detect early progression of VKD. In particular, patients with small neck-shaft angles may be at higher risk of developing VKD, and therefore require more careful follow-up. It is recommended that imaging be performed to evaluate the entire lower extremity. If progression of VKD is anticipated, it is important to consider temporary hemiepiphysiodesis during the growth period to control the progression of the deformity. In conclusion, the present study suggests that MPTA is a factor associated with VKD in patients with MO. Additionally, the neck-shaft angle is suggested as a factor associated with the development of VKD from an early age. However, the present study did not reveal the specific mechanism of VKD development, and association of VKD with MPTA. To resolve this question, a prospective study with data of the entire lower extremity, including the knee, hip, and ankle joints, in addition to findings of CT and MRI, is needed.

## Acknowledgments

The authors would like to thank Enago (www.enago.jp) for the English language review. The authors would like to thank Ms. Yoko Kawai, Tae Naganuma, and Mitsuko Yoshino for the secretarial assistance.

## Author contributions

**Conceptualization:** Kan Ito, Yoshihiro Nishida.

**Data curation:** Kan Ito.

**Formal analysis:** Kan Ito, Kazuki Nishida.

**Investigation:** Kan Ito.

**Methodology:** Kan Ito.

**Project administration:** Yoshihiro Nishida.

**Supervision:** Yoshihiro Nishida, Shiro Imagama.

**Writing – original draft:** Kan Ito.

**Writing – review & editing:** Yoshihiro Nishida, Kunihiro Ikuta, Hiroshi Urakawa, Tomohisa Sakai, Hiroshi Koike.
